# Clinical and radiographic outcomes of oblique lumbar interbody fusion with anterolateral screw and rod instrumentation in osteopenia patients: a retrospective study

**DOI:** 10.1186/s12891-023-06873-1

**Published:** 2023-09-26

**Authors:** Renjie Li, Yijie Liu, Yi Zhu, Minhua Lu, Weimin Jiang

**Affiliations:** 1 Department of Orthopedic Surgery, Dushu Lake Hospital Affiliated to Soochow University, Medical Center of Soochow University, Suzhou Dushu Lake Hospital, Suzhou, Jiangsu, 215123 China; 2 Department of Orthopedic Surgery, The first affiliated hospital of Soochow University, Suzhou, 215006 Jiangsu China

**Keywords:** Oblique lumbar interbday fusion, Bone mineral density, Osteopenia, Anterolateral instrumentation

## Abstract

**Purpose:**

The purpose of this paper is to evaluate the clinical and radiographic outcomes of oblique lumbar interbody fusion (OLIF) to perform in L4/5 degenerative lumbar spondylolisthesis (DLS) patients who diagnosed with osteopenia.

**Methods:**

From December 2018 to 2021 March, 94 patients were diagnosed with degenerative spondylolisthesis underwent OLIF and divided into two groups with different bone mineral density. Anterolateral screw and rod instrumentation was applied in two groups. The primary outcomes were VAS, JOA and ODI. The secondary outcomes included disc height (DH), cross-sectional height of the intervertebral foramina (CSH), cross-sectional area of the dural sac (CSA), lumbar lordorsis (LL), pelvic titlt (PT), pelvic incidence (PI) and sacrum slop (SS).

**Results:**

All patients finished at least 1 years follow-up with 21.05 ± 4.42 months in the group A and 21.09 ± 4.28 months in the group B. The clinical symptoms were evaluated by VAS, JOA and ODI and 94 patients showed good outcomes at final follow-up (*P* < 0.05), with significant increases in DH, CSH and CSA. In group A, DH increased from 8.54 ± 2.48 to 11.11 ± 2.63 mm, while increased from 8.60 ± 2.29 to 11.23 ± 1.88 were recorded in group B. No statistical difference was found in DH between the two groups (*P* > 0.05). The cage subsidence was 1.14 ± 0.83 mm in group A and 0.87 ± 1.05 mm in group B (*P* > 0.05). There was no significant difference in the adjusted parameters of spino-pelvic between two groups (*P* > 0.05).

**Conclusion:**

Oblique lumbar interbody fusion with anterolateral screw and rod instrumentation is feasible to be performed in osteopenia patients who diagnosed with degenerative spondylolisthesis.

**Supplementary Information:**

The online version contains supplementary material available at 10.1186/s12891-023-06873-1.

## Introduction

The oblique anterolateral retroperitoneal pre-psoas approach for oblique lumbar interbody fusion (OLIF) is an emerging procedure that has progressively been used by spine surgeons [1, 2]. Oblique lumbar interbody fusion was first reported by Mayer [3], but it was not popularized at that time. In 2006, Ozgur and Pimenta [4] reported extreme lateral interbody fusion (XLIF), a technology similar to direct lumbar interbody fusion (DLIF). Despite with the neuro-physiological monitoring, XLIF/DLIF still has a high incidence of lumbar plexus injury, such as lower limb pain and numbness.

Since its introduction in 2012 by Silvestre [5], OLIF has been used increasingly as an alternative to conventional anterior or posterior procedures, with favorable clinical results and few early complications [5–7]. It was firstly applied only for indications such as degenerative spondylolisthesis and then the indications were broadened for conditions previously regarded as relative contraindications, such as spinal tuberculosis and ankylosing spondylitis.

In the past years, severe osteoporosis is considered as contraindication of oblique lumbar interbody fusion [8]. Tempel [9] found that patients with BMD T < 1.0 had a significantly higher risk of cage subsidence. Oh [10] demonstrated that osteoporosis was positively correlated with the cage subsidence. We used to define osteopenia as T value between − 1.0 and − 2.5. Meanwhile, osteoporosis is diagnosed when T value is less than − 2.5. Supplemental posterior pedicle screws instrumentation is regarded as the golden standard for patients with osteoporosis. Posterior pedicle screw instrumentation was used to apply for OLIF surgery, with disadvantages of time-consuming and expensive expenses [11, 12]. Posterior pedicle screw with vertebroplasty could be a good choice for patients with severe osteoporosis (BMD<-2.5). OLIF combined with anterolateral screw-rod fixation applied through the same surgical corridor in patients with normal bone mineral density (BMD>-1), which has the potential to provide efficient spinal stability and minimize the blood loss, operation time and cost.

In recent years, several studies [13, 14] have proved the effectiveness of oblique lumbar interbody fusion (OLIF) combined with supplemental anterolateral screw and rod instrumentation. Yet, there was no report demonstrated that whether bone mineral density (BMD) has an effect on this fixation method. The purpose of this paper is to evaluate the efficacy of anterolateral fixation performed in osteopenia patients who diagnosed with degenerative spondylolisthesis.

## Methods and materials

### Patient population

A prospectively study of 94 patients (32 males and 62 females) treated by OLIF with supplemental anterolateral screw and rod instrumentation for L4/5 DLS from December 2018 to March 2021. In our opinion, symptom improved after bed rest is the key to perform the surgery. The patients were divided into two groups with different bone mineral density (BMD) which is assessed by dualenergyX-rayabscorptiometry. And it is considered as the gold standard for diagnosing osteoporosis. Group A:-2.5 < BMD<-1;Group B: BMD >-1. Anterolateral screw and rod instrumentation was used in both two groups. 41 patients with a mean age of 68.76 ± 6.76 years old were included in group (A) The remained patients with a mean age of 66.49 ± 7.30 years old were included in group (B) The same type of bone substitutes were used in two groups. For two groups, no difference was found in BMI (24.54 ± 3.31 vs. 24.71 ± 3.07, P > 0.05). Compared with group B, the BMD in group A is relatively smaller (-1.98 ± 0.41 vs. 0.05 ± 0.89, *P* < 0.05). The patients provided informed consent and the study was approved by the Institutional Ethics Committee of Soochow University.

The inclusion criteria were as follows: (1) low back pain with radiating pain of lower limb; (2) pain improved after bed rest; (3) poor effect of conservative treatment for more than 3 months. (4): X-ray showed L4/5 degenerative spondylolisthesis (Meyerding classification of grade I). (5) Patients followed the medical advice of no smoking after surgery. (6) All of them do not take drugs such as corticosteroids, antidepressants or bisphosphonates.

The exclusion criteria included the following: (1) spinal tumor; (2) vertebral fractures of the lumbar spine; (3) severe lumbar spondylolisthesis(Meyerding classification of grade II,III); (4) congenitalspinal deformities; (5) severe spinal stenosis, bony stenosis and severe facet hypertrophy (6) endplate damage during the operation; (7) revision surgery; (8) adjacent segmental disease. The patient’s preoperative data and operative details are shown in Table 1.

**Table 1:** Demographic data of patients

	Group A(N = 41)	Group B(N = 53)	P value	Cohen’s d
Age (years)	68.76 ± 6.76	66.49 ± 7.30	0.131	0.322
Sex, M/F (n)	7/34	25/28	0.002*	--
Height(m)	1.62 ± 0.08	1.63 ± 0.08	0.639	-0.099
Weight (kg)	64.75 ± 10.86	65.99 ± 11.27	0.598	-0.111
BMI (kg/cm^2^)	24.54 ± 3.31	24.71 ± 3.07	0.792	-0.055
BMD (g/cm^2^)	-1.98 ± 0.41	0.05 ± 0.89	**3.36 × 10** ^− 23*^	**-2.917**
Follow up (months)	21.05 ± 4.42	21.09 ± 4.28	0.960	-0.010
Blood loss (ml)	64.51 ± 45.84	59.06 ± 33.77	0.513	0.136
Operative time (min)	70.93 ± 5.61	70.96 ± 5.81	0.977	-0.006
Smoking after surgery (n) Diabetes mellitus (n) COPD (n)	0 6 1	0 7 3	-- 0.824 0.449	-- -- --

*means it was statistically different between the two groups

Group A:-2.5 < BMD<-1;Group B: BMD >-1

BMI = body mas index; BMD = bone mineral density; COPD = chronic obstructive pulmonary diseases

### Data collection and outcome evaluation

All the patients were informed to make a return regularly after surgery. X-ray was taken at each follow-up time. The final X-ray was taken at a little more than 1 year after surgery or even 2 years. CT and MRI were taken at nearly 1 year postoperatively. The mean values are used for the study.

### Functional and radiographic evaluation

The functional evaluation was evaluated by using the Japanese Orthopedic Association (JOA) score, VAS score and Oswestry Disability Index (ODI) which were recorded preoperatively and at each follow-up. The JOA recovery rate (RR)^7^ was defined as follows: RR(%)= (postoperative JOA score- preoperative JOA score)/ (29-preoperative JOA score)×100%. The results were classified into four groups: 75% or more (excellent), 50–74% (good), 25–49% (fair) and 25% or less represented poor.

**Fig. 1:**
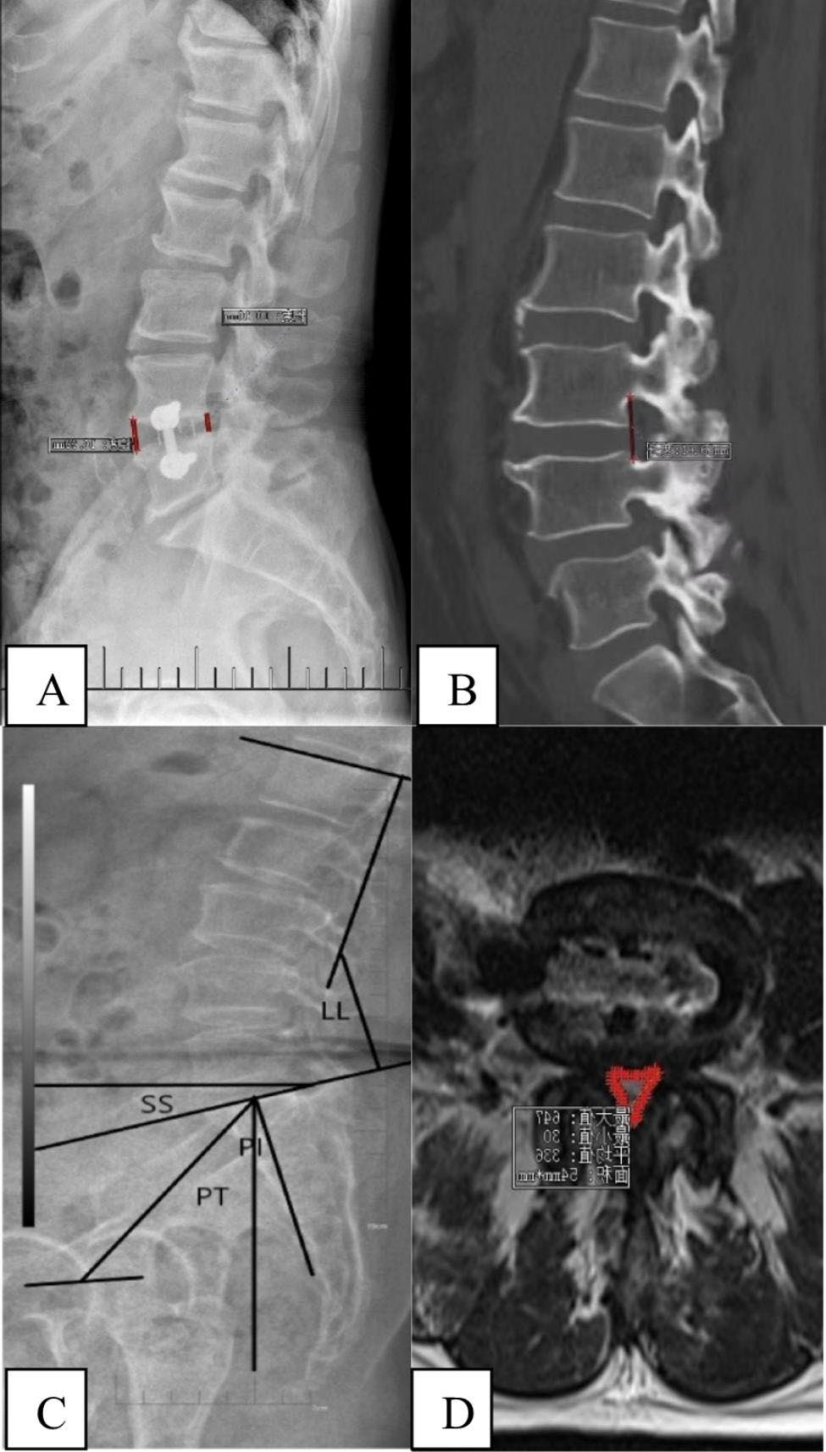
(**A**) Postoperative disc height (1/2 sum of height of anterior and posterior interverterbral space, DH); (**B**) Postoperative cross-sectional height of the intervertebral foramina (CSH) (**C**) Spino-pelvic sagittal parameters (pelvic tilt, PT; pelvic incidence PI; sacral slope, SS;Lumbar lordosis, LL); (D)Cross-sectional area of the dural sac(CSA).

The radiological definitions are defined as the following: (1) disc height (DH), is defined as the one half of the sum of height of anterior and posterior intervertebral space in lateral view. (2) cross-sectional height of the intervertebral foramina (CSH), is defined as the distance between the upper and lower margin of foramina on sagittal CT. (3) cross-sectional area of the dural sac (CSA), was defined as the area of spinal canal were evaluated by T2-weighted axial MRI. (4) Pelvic incidence (PI) was formed by the line vertical to the midpoint of sacral plate and the line between the midpoint of the sacral plate of S1 and the center of the hip joint. (5) Pelvic tilt (PT) was defined as the angle between the line connecting the midpoint of the sacral endplate with the axis of femoral heads and the vertical line. (6) Sacral slope (SS) was defined as the angle formed between the upper endplate of S1 and the horizontal line. (7) Lumbar lordosis (LL) was defined as the angle between the upper endplate of the L1 and S1 vertebra using the Cobb method. (Fig. 1)

### Operative procedure

**Fig. 2:**
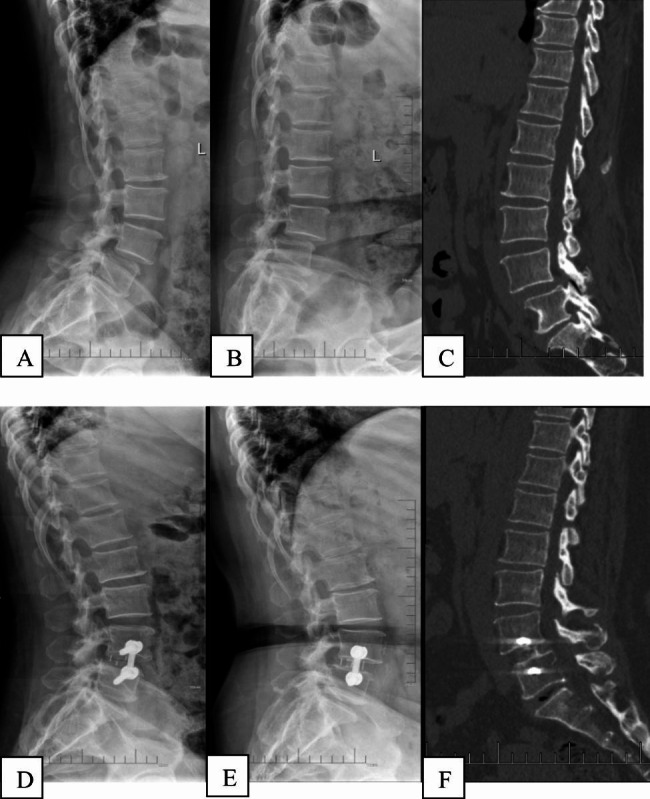
Images of a 56-year old female patient with normal bone density. (**A**)-(**C**). Preoperative lateral radiograph and CT show the L4/5 degenerative spondylolisthesis. (**D**)-(**F**). Postoperative lateral radiograph and CT showed the surgery provided reduction and fixation with satisfactory clinical results and relatively high fusion rate. (D:Postop 3d; E、F: follow-up at 1 year postoperatively)

**Fig. 3:**
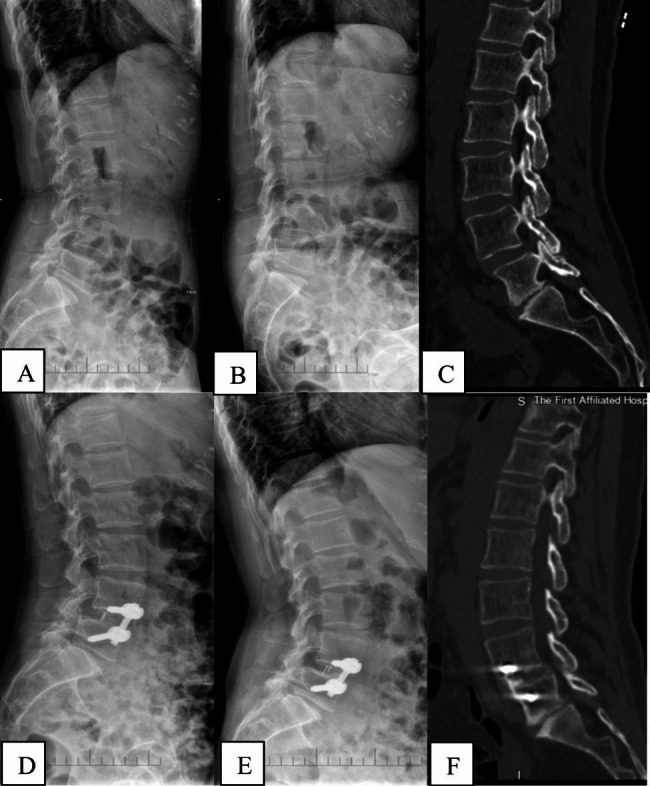
Images of a 59-year old female patient who was diagnosed with osteopenia. (**A**)-(**C**).Preoperative lateral radiograph and CT show the L4/5 degenerative spondylolisthesis. (**D**)-(**F**). Postoperative lateral radiograph and CT showed the surgery provided reduction and fixation with satisfactory clinical results and relatively high fusion rate. (D:Postop 3d; E、F: follow-up at 1 year postoperatively)

The surgery was performed by the same senior surgeon. Following general anesthesia via nasotracheal intubation, the patient was carefully placed in a right side lateral decubitus position on a carbon table. Under C-arm fluoroscopic guidance, the target disc was confirmed. A 4 cm skin incision was made at the left abdomen, and serial dissection of three layers of abdominal muscles-the external oblique, internal oblique, and transversalis abdominis muscles-was performed by blunt dissection. Then the surgeon uses the index finger to confirm the anterior border of the psoas muscle, sliding from the quadratus lumborum muscle to reach there. The retroperitoneal space was accessed by blunt dissection, and the peritoneal content was mobilized anteriorly. Neuromonitoring wasn’t needed since the procedure was not trans-psoas. A Kirschner wire was placed into the disc space from the anterolateral corner to identify the target disc space again. Sequential dilators were placed over the Kirschner wire. Place the self-retaining retractor under illumination to ensure the operation field. A window was made in the annulus fibrosis and the nucleus pulposus was removed with the nucleus pulposus clamp, then the disc material including the remaining cartilaginous end-plate was excised sequentially. The above procedures were done step by step under C-arm fluoroscopic guidance. After that, an appropriate-sized cage filled with allogeneic bone with BMP-2 was inserted orthogonally in a press-fit fashion into the disc spaces. Anterolateral screws were placed into the vertebral bodies. The entrance points of screws were proximal to the adjacent endplate. A rod was then applied and fastened. The wound was closed in the usual way after removing the OLIF retractor systems. Drainage catheter was not needed. Patients were informed that smoking was forbidden after surgery (Figs. 2 and 3). All patients were asked to take anti-osteoporosis drugs regularly, such as calcium tablet and vitamin D.

### Statistical analysis

Data analysis was performed by Microsoft Excel 2016 (Microsoft, Seattle, WA) and SPSS 19.0(SPSS, Chicago, IL, USA). The student’s t-test was used to analyze the numerical data obtained within a normal distribution. The results were presented as the mean ± standard deviation. Pre- and postoperative clinical and radiographic outcomes were compared statistically by using the paired t test. The results were considered significant when P was lee than 0.05 or Cohen’s d was more than 1.

## Results

### Perioperative parameters

There is no significant difference in the operative details such as blood loss and operative time (64.51 ± 45.84 vs. 59.06 ± 33.77; 70.93 ± 5.61 vs.70.96 ± 5.81, *P* > 0.05 ). (Table 1)

### Functional outcomes

The corresponding JOA scores increased significantly from 7.51 ± 0.0.70 preoperatively to 23.78 ± 4.41 postoperatively in the group A and from 7.32 ± 0.75 preoperatively to 24.53 ± 4.18 postoperatively in the group B. No significant difference was found in final JOA scores between the two groups (*P* > 0.05). Compared with the preoperative, significant functional improvement was shown through the decreased ODI score after surgery for both groups (-38.51 ± 13.01 for group A vs.-42.43 ± 12.09 for group B). Low back pain, assessed by VAS, showed a -4.34 ± 0.98 and − 4.09 ± 1.0 improvement at final follow-up in two groups, respectively (*P* > 0.05). Pain in legs, assessed by VAS, showed a -5.29 ± 1.27 and − 4.92 ± 0.97 improvement at final follow-up in two groups(*P* > 0.05). The group A and B showed results of ‘good’ at 3 days postoperatively and ‘excellent’ at final time (Table 2).

**Table 2:** Comparison between Preoperative and postoperative clinical outcomes

	Preop		Postop 3d		Final Follow-up	Final FU-Preop		P value	Cohen’s d
						Value	95%CI		
Group A									
Back pain (JOA,0–29 points)		7.51 ± 0.70		22.59 ± 3.38	23.78 ± 4.41	1.35 16.27 ± 1.35		**4.55 × 10** ^**− 37**^	**-5.153**
Recovery (RR, 0-100%)		--		0.70 ± 0.15	0.76 ± 0.20	-- --		--	--
Disability (ODI, 0-100%)		55.63 ± 9.30		21.88 ± 5.91	17.12 ± 8.11	3.98 -38.51 ± 3.98		**1.77 × 10** ^**− 32**^	**4.413**
Back pain (VAS,0–10 points)		6.83 ± 0.76		3.05 ± 0.73	2.49 ± 0.77	0.299 -4.34 ± 0.299		**5.29 × 10** ^**− 40**^	**5.672**
Leg pain (VAS,0–10 points)		7. 05 ± 1.06		2.88 ± 0.67	1.76 ± 0.53	0.389 -5.29 ± 0.389		**2.11 × 10** ^**− 43**^	**6.322**
Group B									
Back pain (JOA,0–29 points)		7.32 ± 0.75		23.02 ± 2.90	24.53 ± 4.18	1.147 17.21 ± 1.147		**6.26 × 10** ^**− 52**^	**-5.728**
Recovery (RR, 0-100%)		--		0.72 ± 0.14	0.79 ± 0.19	-- --		--	--
Disability (ODI, 0-100%)		59.58 ± 8.32		22.36 ± 4.72	17.15 ± 8.35	3.255 -42.43 ± 3.255		**2.95 × 10** ^**− 47**^	**5.093**
Back pain (VAS,0–10 points)		6.85 ± 0.76		3.04 ± 0.75	2.75 ± 0.67	0.268 -4.09 ± 0.268		**9.09 × 10** ^**− 52**^	**5.705**
Leg pain (VAS,0–10 points)		7.11 ± 0.88		2.87 ± 0.65	2.19 ± 0.80	0.261 -4.92 ± 0.261		**1.07 × 10** ^**− 52**^	**5.838**

*means it was statistically different from the preoperative

Preop = preoperative; Postop = postoperative; FU = Follow up

JOA = Japanese Orthopaedic Association

ODI = Oswestry Disability Index

RR = JOA recovery rate= (Postop JOA-Preop JOA)/(29-preop JOA)

### Complications

In group A, six cases of pain in front of thigh and one case of abdominal distension were observed in patients. In group B, two cases of pain in front of thigh, one case of abdominal distension ad one case of abdominal distension combined with external thigh skin numbness were observed in patients.

### Radiological outcomes

Compared with the preoperative, prominent radiologic improvement was shown through the increased DH, CSA and CSH after surgery. The mean DH increased significantly from the preoperative evaluation to the last follow-up, with an increase from 8.54 ± 2.48 mm preoperatively to 12.26 ± 2.66 mm postoperatively in the group A and from 8.60 ± 2.29 mm to 11.23 ± 1.88 mm postoperatively in the group B. Two cases of cage subsidence were observed in the group A. Compared with first postoperative follow up after surgery, decreased DH at final follow-up were 1.14 ± 0.83 and 0.87 ± 1.05 in two groups, respectively. No statistical difference was found in cage subsidence between the two groups (*P* > 0.05). Statistically differences were observed in two groups in the increased CSH (P < 0.05) while no difference was seen in the increased CSA (P < 0.05, Cohen’d < 1). Both two groups achieved significant improvements in LL after surgery. No significant difference was observed in the spino-pelvic sagittal balance parameters between the two groups after surgery (*P* > 0.05) (Tables 3 and 4).

**Table 3:** Comparison between Preoperative and postoperative radiographic data

	Preop	Postop 3d	Final Follow-up	Final FU-Preop		P value	Cohen’s d
				Value	95%CI		
Group A							
DH(mm)	8.54 ± 2.48	12.26 ± 2.66	11.11 ± 2.63	0.602 2.58 ± 0.602		**2.19 × 10** ^**− 5**^	**-1.005**
CSH(mm)	14.57 ± 2.85	--	17.64 ± 3.25	0.829 3.07 ± 0.829		**2.38 × 10** ^**− 5**^	**-1.004**
CSA(mm^2^)	74.20 ± 37.54	--	104.05 ± 33.11	7.246 29.85 ± 23.67		**0.0003**	-0.843
LL (°)	21.83 ± 14.82	32.10 ± 11.14	30.49 ± 10.90	3.099 8.66 ± 3.099		**0.004**	0.666
PT (°)	21.49 ± 11.09	22.44 ± 10.57	22.27 ± 10.99	3.883 0.78 ± 3.883		0.753	-0.071
PI (°)	50.37 ± 9.15	53.07 ± 9.78	52.59 ± 10.76	3.614 2.22 ± 3.614		0.323	-0.222
SS (°)	29.17 ± 10.05	31.02 ± 9.36	30.29 ± 10.82	3.861 1.12 ± 3.861		0.632	-0.107
Group B							
DH(mm)	8.60 ± 2.29	12.10 ± 2.14	11.23 ± 1.88	0.674 2.63 ± 0.674		**4.48 × 10** ^**− 9**^	**-1.256**
CSH(mm)	14.76 ± 2.18	--	18.01 ± 2.46	0.774 3.26 ± 0.774		**1.35 × 10** ^**− 10**^	**-1.399**
CSA(mm^2^)	59.31 ± 34.82	--	84.77 ± 36.12	6.585 25.46 ± 6.585		**3.99 × 10** ^**− 4**^	-0.718
LL (°)	22.62 ± 14.73	34.38 ± 12.68	31.92 ± 12.39	3.599 9.30 ± 3.599		**1.756 × 10** ^**− 16**^	-0.684
PT (°)	20.49 ± 10.44	21.23 ± 9.81	21.02 ± 12.11	3.631 0.53 ± 3.631		0.812	-0.047
PI (°)	49.75 ± 8.57	50.91 ± 9.63	51.21 ± 9.10	2.942 1.45 ± 2.942		0.404	-0.164
SS (°)	29.26 ± 12.14	29.68 ± 10.51	30.19 ± 11.89	4.150 0.92 ± 4.150		0.696	-0.077

*means it was statistically different from the preoperative

DH = disc height; CSH = cross-sectional height of the intervertebral foramina; CSA = cross-sectional area of the dural sac;

LL = lumbar lordosis; PT = pelvic tilt; PI = pelvic incidence; SS = sacrum slop

**Table 4:** Comparison of clinical and radiological data in two groups

	Group A	Group B	P value	Cohen’s d
ΔBack pain (JOA,0–29 points)	16.27 ± 4.41	17.21 ± 4.26	0.304	0.946
ΔDisability (ODI, 0-100%)	-38.51 ± 13.01	-42.43 ± 12.09	0.139	0.312
ΔBack pain (VAS,0–10 points)	-4.34 ± 0.98	-4.09 ± 1.0	0.237	0.253
ΔLeg pain (VAS,0–10 points)	-5.29 ± 1.27	-4.92 ± 0.97	0.119	0.327
Cage subsidence (mm)	-1.14 ± 0.83	-0.87 ± 1.05	0.186	-0.408
Cage subsidence (n)	2	3	0.621	--
Type of Modic changes (n) Type I Type II Type III	0 6 1	2 3 0	--	--
LL loss (°)	-1.61 ± 2.72	-2.45 ± 2.98	0.166	-0.294
PI loss (°)	-0.49 ± 9.61	-0.30 ± 6.20	0.634	-0.024
PT loss (°)	-0.17 ± 5.75	-0.21 ± 11.53	0.985	0.0044
SS loss (°)	-0.73 ± 8.25	0.51 ± 11.96	0.575	-0.121
Screw loosen (n)	0	0	--	--

Δ= value of (final follow-up-preop follow up)

loss = value of (final follow-up-postop 3d)

Cage subsidence= (DH at 3 days postopeartively-DH at final follow-up postopeartively) > 3 mm

## Discussion

Osteoporosis has become an important health problem for middle-aged and elderly people. Zachary [9] suggested that the rate of graft subsidence following LLIF is inversely related to bone mineral density. The negative effect in patients with poor bone mineral density has motivated spine surgeons to investigate various pharmacologic methods, such as bis-phosphonates, to maximize bone quality in those kinds of patients. The measurement of preoperative imaging parameters, pedicle screw with vertebroplasty, intraoperative protection of bone endplate and adjustment of bone metabolism postopeartively can effectively improve the imaging results and clinical efficacy after operation.

Since its first introduction in 2012, oblique lumbar interbody fusion (OLIF) has evolved and led to improved outcomes in properly selected patients. Posterior pedicle screw instrumentation was routinely used for stabilization after the OLIF procedure. Traditional bilateral internal fixation is more stable than unilateral fixation in axial rotation and lateral curvature [15]. However, a study [16] has shown that over rigid fixation lead to stress shielding at the fusion segment, resulting in accelerated degeneration of adjacent segments, as well as bone graft absorption of corresponding vertebral bodies. A vitro biomechanical study [17] demonstrated that unilateral pedicle screw fixation combined with a single cage can restore torsional stiffness and other spinal stability indexes. A clinical study [18] showed that unilateral internal fixation decreased the operation time, blood loss and the cost of implants, while no significant difference was found in the clinical efficacy and fusion rate with bilateral internal fixation. Liu et al. [14] performed a prospective study to assess outcomes in 14 patients with lumbar degenerative diseases and reported that patient underwent OLIF and anterolateral screw-rod instrumentation led to a satisfactory effect, which proved that it minimized the operaton time, iatrogenic injuries and medical cost and has the potential to provide efficient spinal segment stability. In this study, a total of 94 patients underwent OLIF combined with anterolateral screw-rod instrumentation and all of them achieved good clinical outcomes at final follow-up.

Cage subsidence is one of the common postoperative indicators that needed to be paid attention. Tokuhashi [19] suggested that the cage subsidence is inevitable on imaging and the subsidence mainly occurred within 1 year after operation, compared with the first postoperative X-ray. The loss of height of intervertebral space of fusion segment was seen at the last follow-up. The degree of cage subsidence increased with time before it achieved spinal stability. Le [20] found that patients with clinical symptoms accounted for 2.1% of imaging subsidence and whether there is a correlation between the cage subsidence and symptoms is unknown. However, severe cage subsidence will lead to fusion failure, spinal sagittal imbalance and Basstrup syndrome [21]. Severe cage subsidence is accompanied by obvious screw loosening and excessive loss of intervertebral space height, resulting in intervertebral foramen stenosis, recurrence or aggravation of low back pain. Zeng et al. [22] performed a study to analyze the early complications OLIF and showed that 18 cases of cage sedimentation or cage transverse shifting in the OLIF stand-alone group. In this study, there were totally 5 cases of cage subsidence in two groups. Similar to the normal BMD group (group B), the height of intervertebral space and lumbar lordosis in the osteopenia group (group A) were significantly higher than those before operation. At the final follow-up, the height of intervertebral space decreased compared with 3 days after operation, but it was still higher than that before operation, and there was no corresponding clinical symptom occurred. Spinal stability is the main reason of early symptom improvement after surgery, and the poor clinical effect is closely related to cage subsidence. Yang [10] demonstrated that hyperextension of intervertebral space increases the stress of fusion segment, accelerating the occurrence of subsidence and degeneration of adjacent segment. In this study, the disc height is mainly corrected by gradual expansion of the intervertebral space. After inserting the cage, the intervertebral space is pressurized by elastic retraction, avoiding the excessive pressure of intervertebral space and decreasing the risk of loss of intervertebral height. According to our experience, we hold the view that the location of vertebral screw is the junctional area of 1/2 and 1/3 of the vertebral body, closing to the upper and lower endplates, where provides the most rigid fixation for spinal column. There was no significant difference in postoperative lumbar lordosis both in group A and B, compared with that before operation. Excessive correciton of lumbar lordosis will lead to greater compression force in the posterior part ot endplates. The interface area between the cage and the endplate is decreased because of excessive expansion of the anterior intervertebral space, increasing the accidence of cage subsidence. Therefore, excessive correction of the lumbar lordosis should be avoided in patients with osteopenia. Glassman [23] demonstrated that the positive sagittal balance was the most reliable predictor of clinical symptoms. Higher PT means that the pelvis is in a backward state, increasing the tension of lumbosacral ligaments and muscles, which could be accounted for low back pain. In this study, no significant difference was found in spino-pelvic sagittal parameters between 3 days after operation and the final follow-up (*P* > 0.05). It demonstrated that anterolateral screw and rod instrumentation maintained spinal sagittal balance both in osteopenia and normal BMD patients. Besides, we found that improvement in sacrum slope (SS) or pelvic tilt (PT) were limited. The poor effect on mismatch of pelvic incidence-lumbar lordosis highlights the limitation of OLIF cage to achieve sagittal balance.

This study had some limitations. The group size was relatively small and therefore this study may be underpowered to detect many changes. The influence of age-related natural degeneration of spine was not excluded. Longer follow-ups are needed to confirm the results we obtained in this study.

## Conclusion

OLIF is a relatively safe alternative to traditional posterior approaches and our study demonstrated that OLIF with anterolateral screw and rod instrumentation is feasible to perform in osteopenia patients who diagnosed with degenerative spondylolisthesis.

### Electronic supplementary material

Below is the link to the electronic supplementary material.

Supplementary Material 1

Supplementary Material 2

## Data Availability

The datasets used and/or analyzed during the current study available from the corresponding author on reasonable request.

## Abbreviations

OLIF: Oblique lumbar interbody fusion
XLIF: extreme lateral interbody fusion
DLIF: direct lumbar interbody fusion
DH: disc height
CSH: cross-sectional height of the intervertebral foramina
CSA: cross-sectional area of the dural sac
JOA: Japanese Orthopaedic Association Scores
ODI: Oswestry Disability Index
