# Supplementary material for: Clinical and radiographic outcomes of oblique lumbar interbody fusion with anterolateral screw and rod instrumentation in osteopenia patients: a retrospective study
Source: BMC Musculoskelet Disord. 2023 Sep 26;24:760. doi: 10.1186/s12891-023-06873-1 (PMC10521488; doi:10.1186/s12891-023-06873-1)
Supplement: Supplementary file 1 [file 12891_2023_6873_MOESM2_ESM.docx]

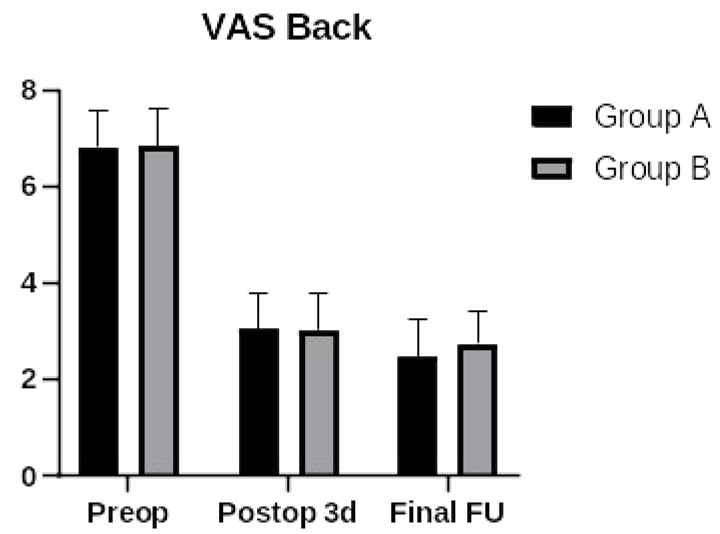
VAS Back
8
6
4
2
0
Preop
Postop 3d
Final FU
Group A
Group B

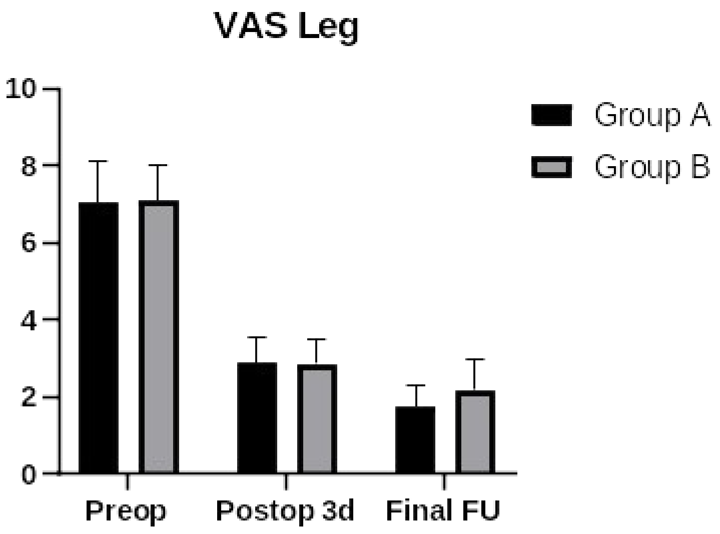
VAS Leg
10
8
6
4
2
0
Preop
Postop 3d
Final FU
Group A
Group B

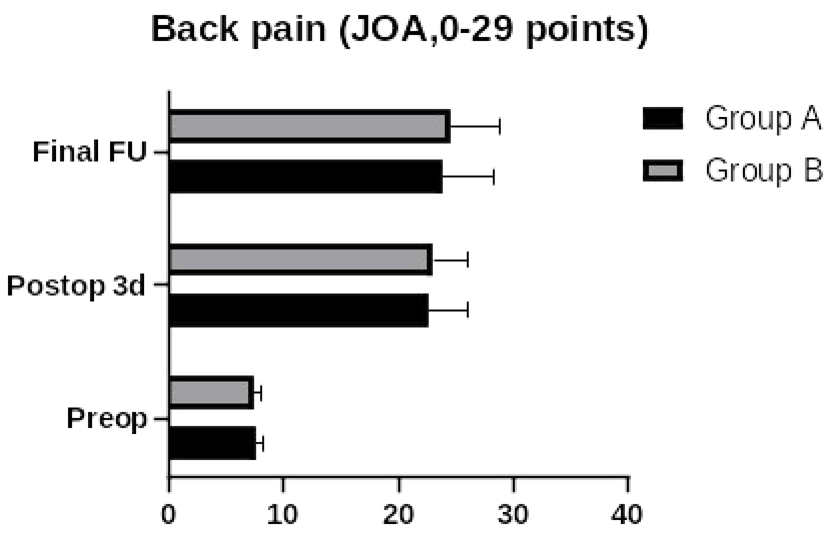
Back pain (JOA,0-29 points)
Final FU
Postop 3d
Preop
0
10
20
30
40
Group A
Group B

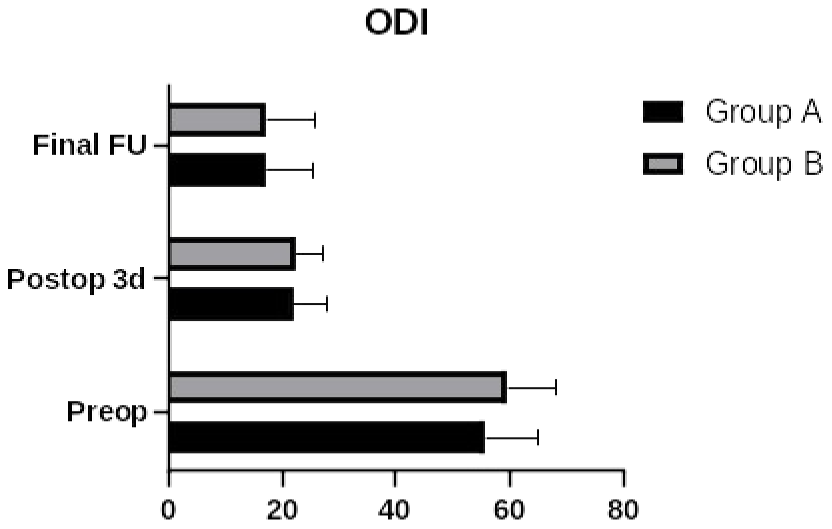
ODI
Group A
Group B
Final FU
Postop 3d
Preop
0
20
40
60
80

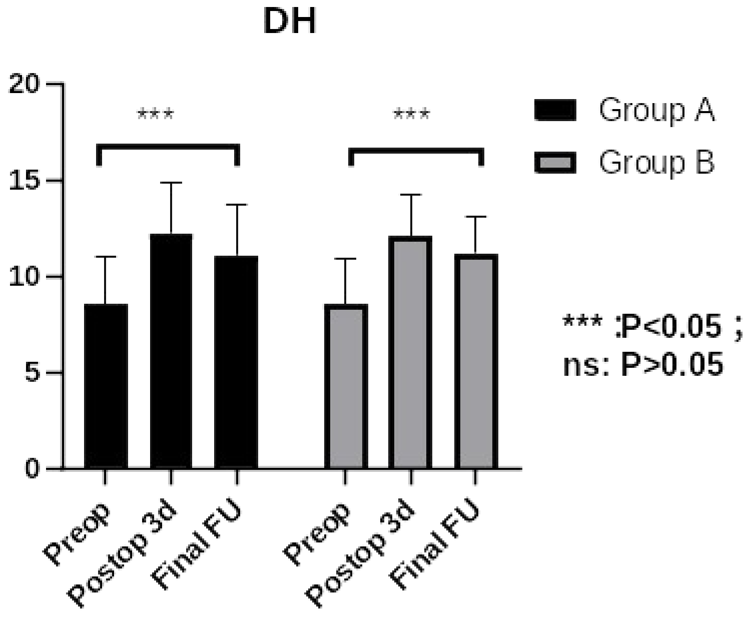
DH
Group A
Group B
***
***
*** :P<0.05 ;
ns: P>0.05
20
15
10
5
0
Preop
Postop 3d
Final FU
Preop
Postop 3d
Final FU

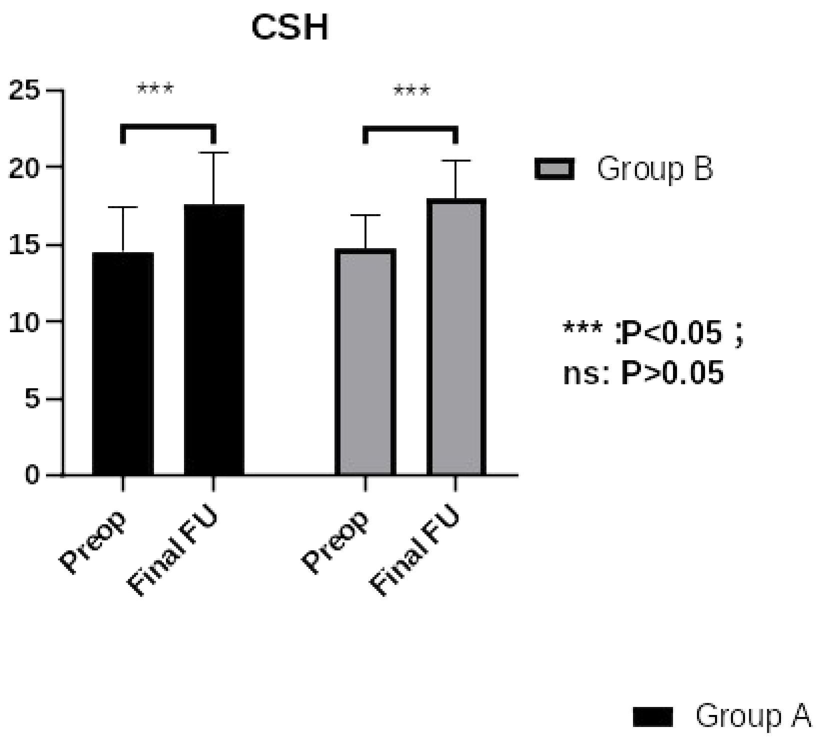
CSH
25
20
15
10
5
0
***
***
Group B
*** :P<0.05 ;
ns: P>0.05
Preop
Final FU
Preop
Final FU
Group A

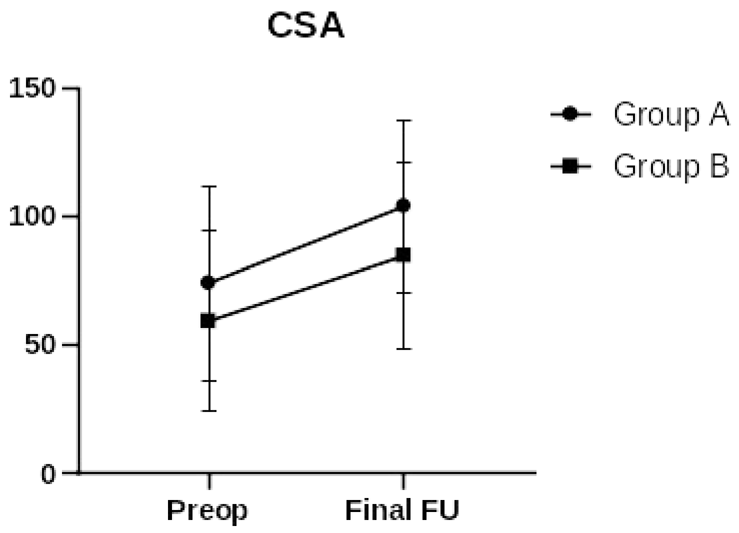
CSA
150
100
50
0
Group A
Group B
Preop
Final FU

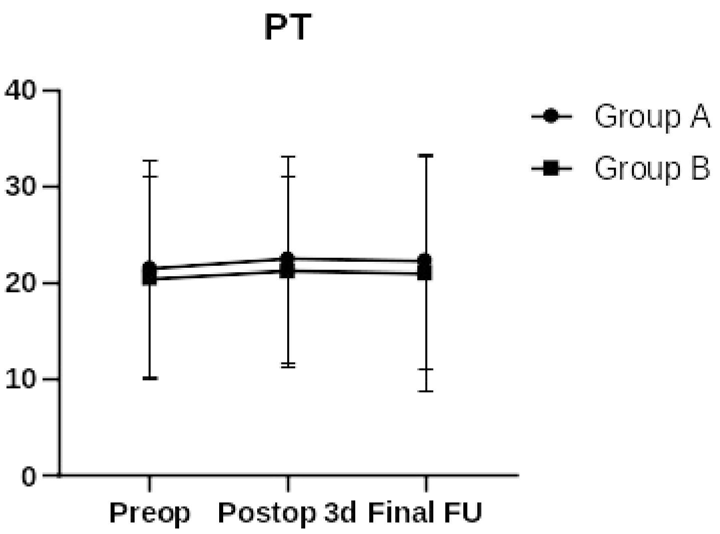
PT
40
30
20
10
0
Preop
Postop 3d
Final FU
Group A
Group B

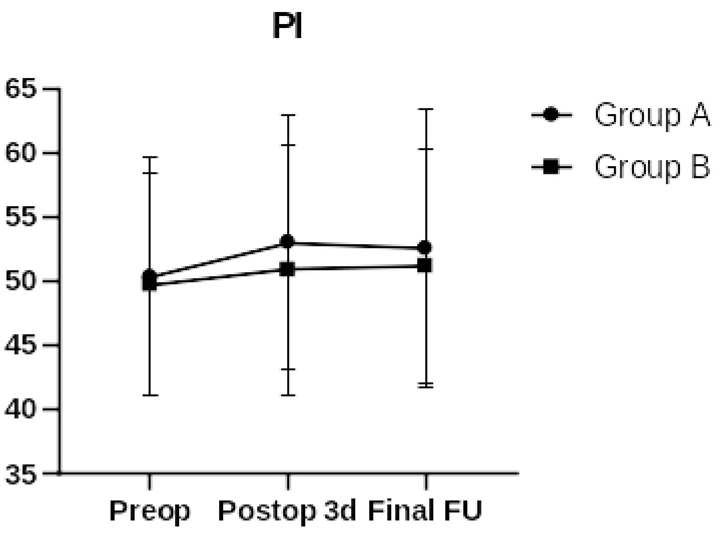
PI
65
60
55
50
45
40
35
Preop
Postop 3d
Final FU
Group A
Group B

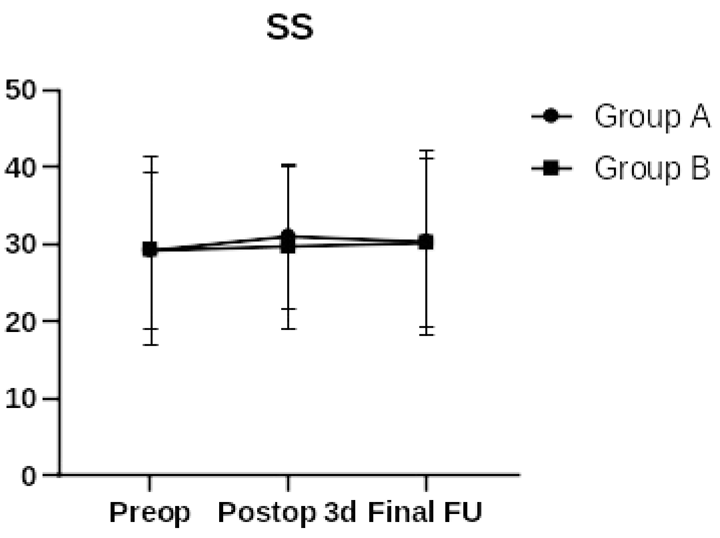
SS
50
40
30
20
10
0
Preop
Postop 3d
Final FU
Group A
Group B

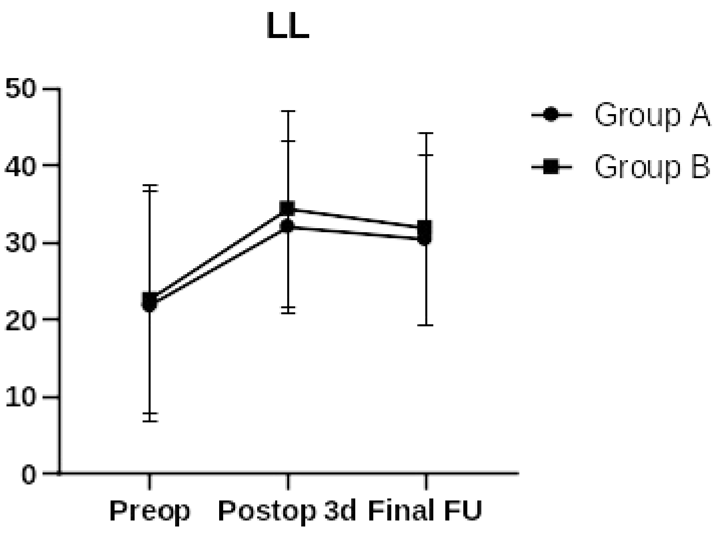
LL
50
40
30
20
10
0
Preop
Postop 3d
Final FU
Group A
Group B
